# Anti-Agglomeration Behavior and Sensing Assay of Chlorsulfuron Based on Acetamiprid-Gold Nanoparticles

**DOI:** 10.3390/nano8070499

**Published:** 2018-07-06

**Authors:** Guangyang Liu, Ruonan Zhang, Lingyun Li, Xiaodong Huang, Tengfei Li, Meng Lu, Donghui Xu, Jing Wang

**Affiliations:** 1 Key Laboratory of Vegetables Quality and Safety Control, Ministry of Agriculture and Rural Affairs of China, Institute of Vegetables and Flowers, Chinese Academy of Agricultural Sciences, Beijing 100081, China; liuguangyang@caas.cn (G.L.); zrn704269188@163.com (R.Z.); lilingyun@caas.cn (L.L.); huangxiaodong@caas.cn (X.H.); 2 College of Life Sciences and Engineering, Hebei University of Engineering, Handan 056021, China; litengfeibeyond@126.com (T.L.); 18230106173@163.com (M.L.); 3 Institute of Quality Standard and Testing Technology for Agro Products, Chinese Academy of Agricultural Sciences, Key Laboratory of Agrifood Safety and Quality, Ministry of Agriculture and Rural Affairs of China, Beijing 100081, China; W_jing2001@126.com

**Keywords:** colorimetric sensing, gold nanoparticle, anti-agglomeration behavior, chlorsulfuron, acetamiprid, agricultural irrigation water

## Abstract

Monitoring of low levels of chlorsulfuron in environmental water samples is important. Although several detection methods have been developed, they all have some drawbacks, such as being time-consuming, requiring expensive instruments and experienced operators, and consuming large volumes of organic solvents. There is an urgent need for a simple, rapid, and inexpensive detection method for chlorsulfuron. Herein, such a method was developed using anti-aggregation of gold nanoparticles (AuNPs) in the presence of acetamiprid in agricultural irrigation water samples. Aggregation of the AuNPs was induced by acetamiprid, and this produced a distinct color change from Bordeaux red to blue. However, the strong hydrogen bonding interaction between chlorsulfuron and acetamiprid could inhibit AuNP aggregation. The effect of chlorsulfuron on the anti-agglomeration behavior of AuNPs was monitored by ultraviolet–visiblespectroscopy (UV-Vis) and the naked eye over a concentration range 0.1–100 mg/L. The detection limit for chlorsulfuron was 0.025 mg/L (signal-to-noise ratio of three). This colorimetric method was successfully applied to the determination of chlorsulfuron in spiked tap water and agricultural irrigation water with satisfactory recoveries (76.3%–94.2%).

## 1. Introduction

Sulfonylurea pesticides, an efficient class of herbicides, are commonly used for weed control in many crops [1]. Chlorsulfuron was the first sulfonylurea herbicide available commercially, and had been widely applied to control grass and broadleaf weeds [2]. Because chlorsulfuron is persistent and tends to migrate in soil and water, residues can be found in rivers and well water, which may harm agricultural crops through irrigation, and even humans and animals through drinking water [3]. Consequently, it is important to establish a simple, rapid, and highly sensitive method to monitor low levels of chlorsulfuron in environmental water samples.

Several analytical methods have been developed for chlorsulfuron analysis, mainly using gas chromatography [4], gas chromatography coupled to mass spectrometry [5], high performance liquid chromatography [6,7], high performance liquid chromatography coupled to mass spectrometry [8], fluorescent chemosensors [9], capillary electrophoresis [10], and immunosensors [11]. Although these analytical methods have high sensitivity and selectivity, they also have some drawbacks, such as being time-consuming, requiring expensive instruments and experienced operators, and consuming large volumes of organic solvents [12,13]. Consequently, a simple, rapid, inexpensive, and highly sensitive analytical method to determine trace amounts of chlorsulfuron in environmental water samples is required.

Gold nanoparticles (AuNPs) have attracted attention in many research areas because of their unique chemical, optical, and electronic properties [14,15]. Their strong surface plasmon resonance endows them with good signal generation and transduction. Many analytical strategies have been developed using AuNPs as signal recognition and transmission units with electrochemistry, fluorescence, bioassay, and colorimetry techniques [16,17,18]. Among these techniques, colorimetric assays based on the aggregation of AuNPs have been widely used for monitoring various pesticide residues, such as atrazine [19,20], triadimenol [21], imidacloprid [22], and dithiocarbamate [23]. However, the aggregation process could be induced by many interferences, and be affected by complex matrices. Recently, many colorimetric sensors based on the anti-aggregation of AuNPs have been developed to enhance the sensitivity and selectivity, and to avoid false positives or incorrect results [24,25,26]. Few studies have exploited anti-aggregation principles to develop an AuNPs-colorimetric assay for chlorsulfuron. Our group has designed the colorimetric sensor to determine the presence of metsulfuron-methyl based on the anti-aggregation of melamine-AuNPs. Although the colorimetric sensor could be used to detect trace amounts of metsulfuron-methyl residues in water samples, the sensor shows instability and poor selectivity because of the high reactivity of melamine molecules. Acetamiprid could induce AuNPs to aggregate and exhibit low reactivity with many interfering substances, which makes it a perfect candidate as the modified molecule in the anti-aggregation process [27]. 

Inspired by anti-aggregation colorimetry, our aim was to establish a simple and sensitive colorimetric assay based on the anti-agglomeration behavior of AuNPs for chlorsulfuron detection in environmental water samples. The aggregation of AuNPs was induced by acetamiprid, and was accompanied by a color change from Bordeaux red to blue. However, strong hydrogen bonding interactions between chlorsulfuron and acetamiprid could inhibit the aggregation of AuNPs, and induce a color shift from blue to red. The changes in absorption spectra could be recorded using an ultraviolet–visiblespectroscopy (UV-Vis) spectrophotometry, and color changes could be observed by the naked eye.

## 2. Materials and Methods 

### 2.1. Chemicals and Apparatus

Chlorsulfuron, acetamiprid, atrazine, and hexazinone were bought from Sigma-Aldrich (St. Louis, MO, USA). NaCl, MgCl_2_, glucose, l-cysteine, and vitamin C were obtained from Aladdin Industrial Corporation (Shanghai, China). Chloroauric acid (HAuCl_4_) was purchased from Sinopharm Chemical Reagent Co., Ltd. (Shanghai, China). All other reagents were of analytical reagent grade.

UV-Vis absorption spectra were recorded on a NanoDrop One^©^ spectrophotometer (Thermo ScientificManufacturer, City, Country, Waltham, MA USA). Transmission electron microscopy was performed using a JEM-200CX transmission electron microscope (TEM, JEOL, Tokyo, Japan). A Fourier transform infrared spectrometer (FT-IR-8400, Shimadzu, Kyoto, Japan) was used to record Fourier transform infrared spectra.

### 2.2. Synthesis of AuNPs

According to the literature [28,29], AuNPs were prepared by a reduction reaction between citrate and HAuCl_4_. All glassware was soaked in aqua regia (*V*_HCl_/*V*_HNO3_ = 3:1), and then cleaned with ultrapure water. HAuCl_4_ aqueous solution (1 mM, 150 mL) was heated at 100 °C for 30 min with vigorous stirring. Trisodium citrate solution (38.8 mM, 150 mL) was then added to the HAuCl_4_ aqueous solution, and the mixture was heated at 100 °C for another 15 min. The color of the solution changed from light yellow to Bordeaux red. After cooling to room temperature, the solution was filtered through a 0.22 µm syringe filter (Millipore, Billerica, MA, USA). The resulting suspension was stored at 4 °C until it was required for use. 

### 2.3. Colorimetric Assay

Typically, 0.2 mL of AuNP solution was mixed with 0.2 mL of acetate buffer solution (10 mM, pH 3.5) and the mixture was incubated for 5 min. Chlorsulfuron (0.2 mL) solutions with different concentrations (0.1, 0.2, 0.5, 1.0, 2.0, 5.0, 10.0, 20.0, 50.0, and 100.0 mg/L) were added, and the solution was incubated for another 5 min. Next, acetamiprid solution (0.1 mL) was added and the final mixture was equilibrated for 20 min. UV-Vis spectra were recorded over the wavelength range 400–750 nm and the color change was observed by both the naked eye and a camera. The change in the absorbance at 523 nm (Δ*A_523_*) was used to determine the concentration of chlorsulfuron. Some common potential interfering substances, such as atrazine, hexazinone, glucose, l-cysteine, vitamin C, Na^+^, and Mg^2+^ were used to investigate the selectivity of this colorimetric method at 5, 10, and 20 mg/L. 

### 2.4. Sample Pretreatment

Ethylenediaminetetraacetic acid (EDTA) was added to the tap water and environmental water samples to a final concentration of 1 mM to remove any heavy metal ions. Then, the solution was filtered through a 0.2 µm syringe filter (Millipore). The prepared suspension was spiked with standard solutions at concentrations of 0.5, 1.0, and 5.0 mg/L. Finally, the colorimetric method was used to determine the chlorsulfuron concentration.

## 3. Results and Discussion

### 3.1. Characterization

**Figure 1:**
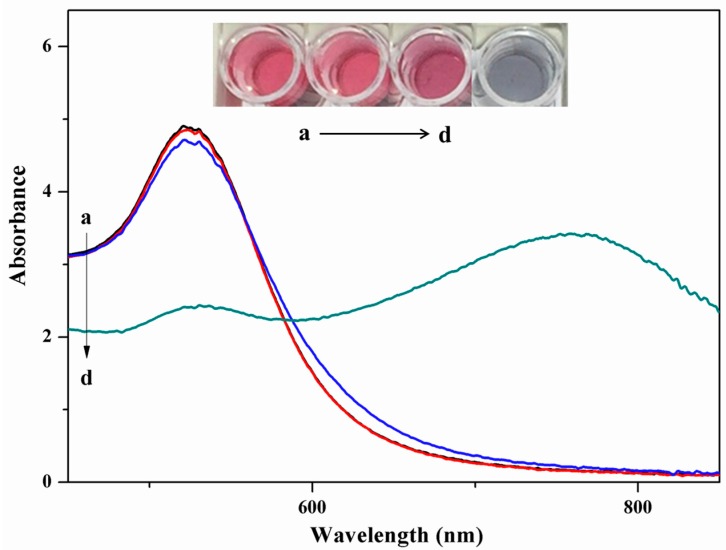
(a) AuNPs (black line), (b) AuNPS with 2.0 mg/L chlorsulfuron (red line), (c) AuNPS with 2.0 mg/L chlorsulfuron and 2.0 mg/L acetamiprid (blue line), (d) AuNPS with 20 mg/L acetamiprid (green line).

Surface plasma resonance absorbance spectra were recorded for AuNPs, AuNPs with 2.0 mg/L chlorsulfuron, AuNPs with 20 mg/L acetamiprid, and AuNPs with 2.0 mg/L chlorsulfuron and 20 mg/L acetamiprid (Figure 1). The AuNPs were red and exhibited strong surface plasmon resonance absorption at about 523 nm. In the presence of chlorsulfuron, the color of the AuNPs was Bordeaux red. After adding 0.1 mL of 20 mg/L acetamiprid to the AuNPs solution, the intensity of the surface plasmon resonance absorption at 523 nm decreased, a new absorption band appeared at 640 nm, and the color changed from Bordeaux red to blue. These results indicated that aggregation of the AuNPs was induced by acetamiprid. However, when chlorsulfuron solutions with different concentrations were added to the AuNPs solution before mixing with acetamiprid, the aggregation of AuNPs induced by acetamiprid was inhibited and the color changed from blue to red. 

**Figure 2:**
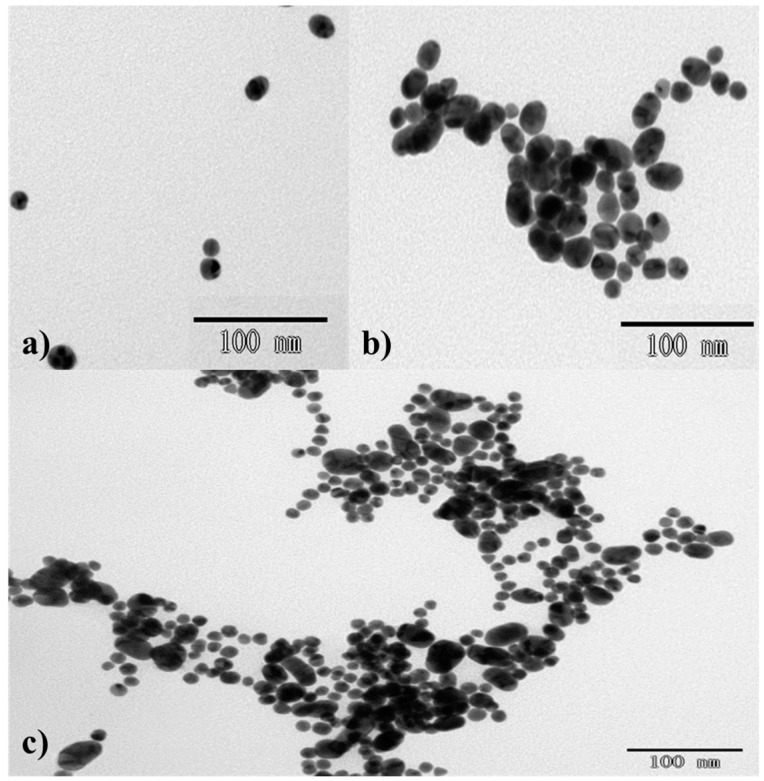
TEM images of (**a**) AuNPs, (**b**) AuNPS with 2.0 mg/L chlorsulfuron and 20 mg/L chlorsulfuron, (**c**) AuNPS with 20 mg/L acetamiprid.

The sizes and morphologies of the AuNPs, AuNPs with 20 mg/L acetamiprid, and AuNPs with 2.0 mg/L chlorsulfuron and 20 mg/L acetamiprid were investigated by transmission electron microscopy (Figure 2). Both the AuNPs and AuNPs with chlorsulfuron were monodisperse, with an AuNP average diameter of about 13 nm. This indicated that chlorsulfuron could not induce AuNP aggregation. In the presence of only acetamiprid, bulk random aggregations of AuNPs were observed. Fewer and smaller aggregates were observed than for AuNPs with 2.0 mg/L acetamiprid (Figure 2b,c), suggesting that formation of a complex between chlorsulfuron and acetamiprid prevented the AuNP aggregation. 

**Figure 3:**
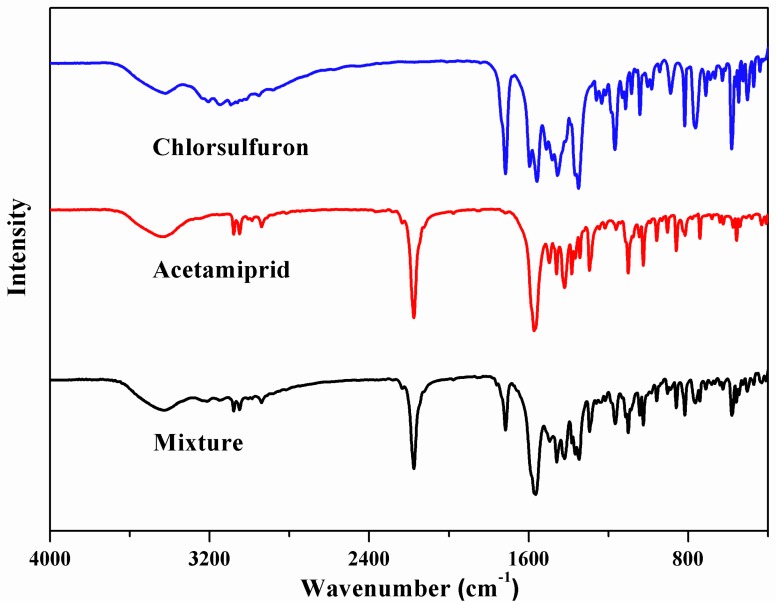
FT-IR spectra of: chlorsulfuron, chlorsulfuron, the complex of acetamiprid and chlorsulfuron.

Fourier transform infrared spectroscopy was used to study the hydrogen bonding interactions between chlorsulfuron and acetamiprid (Figure 3). The spectrum of chlorsulfuron showed adsorption peaks for –R-SO_2_– at 1364 cm^−1^ and 1714 cm^−1^ and a stretching band for –NH– at 1566 cm^−1^. From the spectrum of acetamiprid, the typical peaks observed at 2230 and 1600 cm^−1^ were attributed to the C≡N and C=N groups, respectively. By comparing the spectra of chlorsulfuron, acetamiprid and a complex of chlorsulfuron and acetamiprid, we observed the intensities of both stretching vibrations of the C=N group in acetamiprid and –R-SO_2_– in chlorsulfuron decreased, and the characteristic peak for–NH– in chlorsulfuron shifted. This indicated that hydrogen bonding interactions formed between chlorsulfuron and acetamiprid.

### 3.2. Colorimetric Sensing Mechanism 

**Figure 4:**
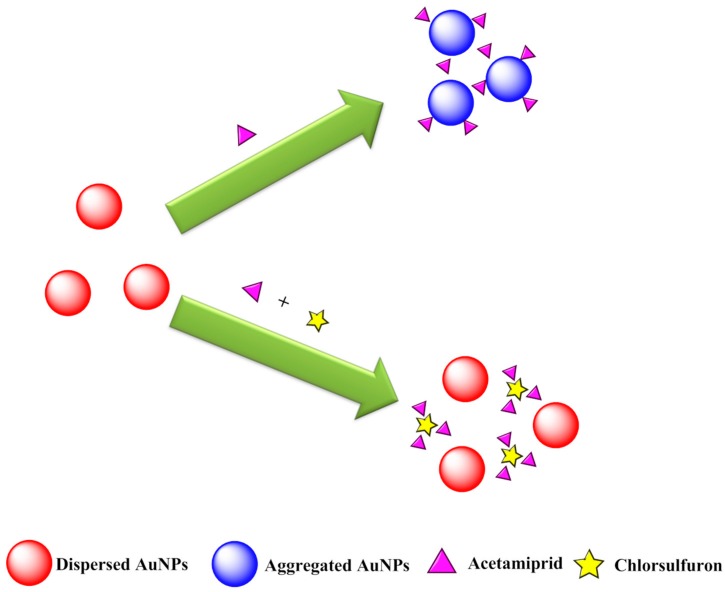
Schematic illustration of Colorimetric sensing of chlorsulfuron based on anti-aggregation of gold nanoparticles in the presence of acetamiprid.

Figure 4 illustrates the colorimetric sensing scheme for chlorsulfuron detection based on the anti-aggregation of AuNPs. The AuNPs solution was wine colored with a strong surface plasmon resonance (SPR) absorption peak at about 523 nm. The aggregation of AuNPs was induced by acetamiprid with an accompanying distinct color change from Bordeaux red to blue. However, strong hydrogen bonding interactions between chlorsulfuron and acetamiprid could inhibit the aggregation of AuNPs. In this sensing system, acetamiprid was used as the aggregation reagent and chlorsulfuron acted as an anti-aggregation reagent. The change in absorbance at 523 nm was linearly related to the change in the chlorsulfuron concentration. Thus, the concentration of chlorsulfuron could be monitored by a UV-Vis spectrophotometer, or even qualitatively by the naked eye. 

### 3.3. Optimization of the Method

Because detection with this colorimetric assay relies on the intensity of the change in color of the solution, it is important to optimize the critical parameters such as the acetamiprid concentration, buffer pH, and contact time, to establish the best analytical conditions.

#### 3.3.1. Effect of the Acetamiprid Concentration

**Figure 5:**
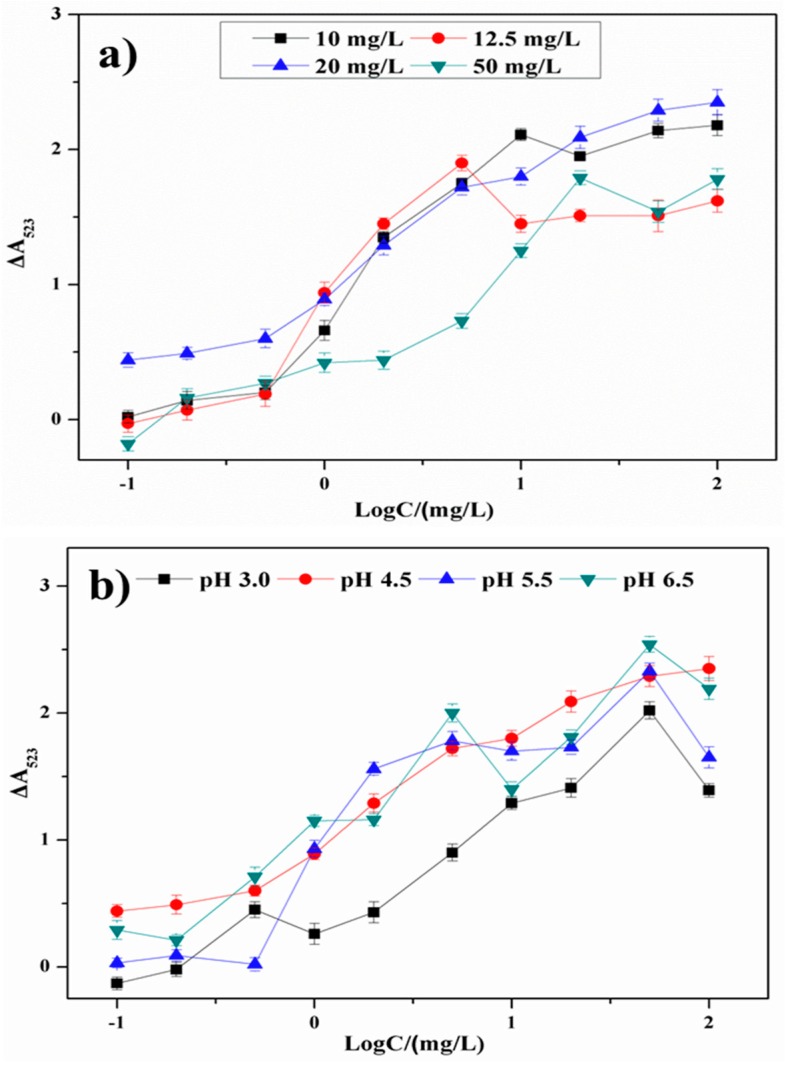
The plots of Δ*A_523_* versus chlorsulfuron concentration under (**a**) different concentration of acetamiprid and (**b**) different pH condition.

To increase the sensitivity of this colorimetric method, an appropriate concentration of acetamiprid needs to be selected to induce AuNP aggregation. The effect of the concentration of acetamiprid on aggregation in the presence of AuNPs and chlorsulfuron was investigated (Figure 5a). An increase in the acetamiprid concentration could cause an increase in the change in absorbance in the presence of different chlorsulfuron concentrations (10–50 mg/L). When the acetamiprid concentration was increased to over 20 mg/L, excess acetamiprid induced too much AuNP aggregation, and this decreased the response of this system in the presence of low chlorsulfuron concentrations. When the acetamiprid concentration was below 12.5 ppm, the response in the presence of high chlorsulfuron concentrations became less sensitive. Therefore, 20 mg/L was chosen as the appropriate acetamiprid concentration for further reactions.

#### 3.3.2. Effect of pH

The pH of the reaction solution will also strongly affect the sensitivity of this sensing system. The effects of different acetate buffer pH values (3.0–6.5) on AuNP anti-aggregation were investigated (Figure 5b). When the pH was below 4.5, the AuNPs more readily aggregated, and the anti-aggregation performance of chlorsulfuron was reduced. When the pH was above 4.5, the response became less sensitive, and this narrowed the linear range of the colorimetric sensing system. Based on above results, pH 4.5 was selected for use in subsequent experiments.

### 3.4. Colorimetric Sensing of Chlorsulfuron

**Figure 6:**
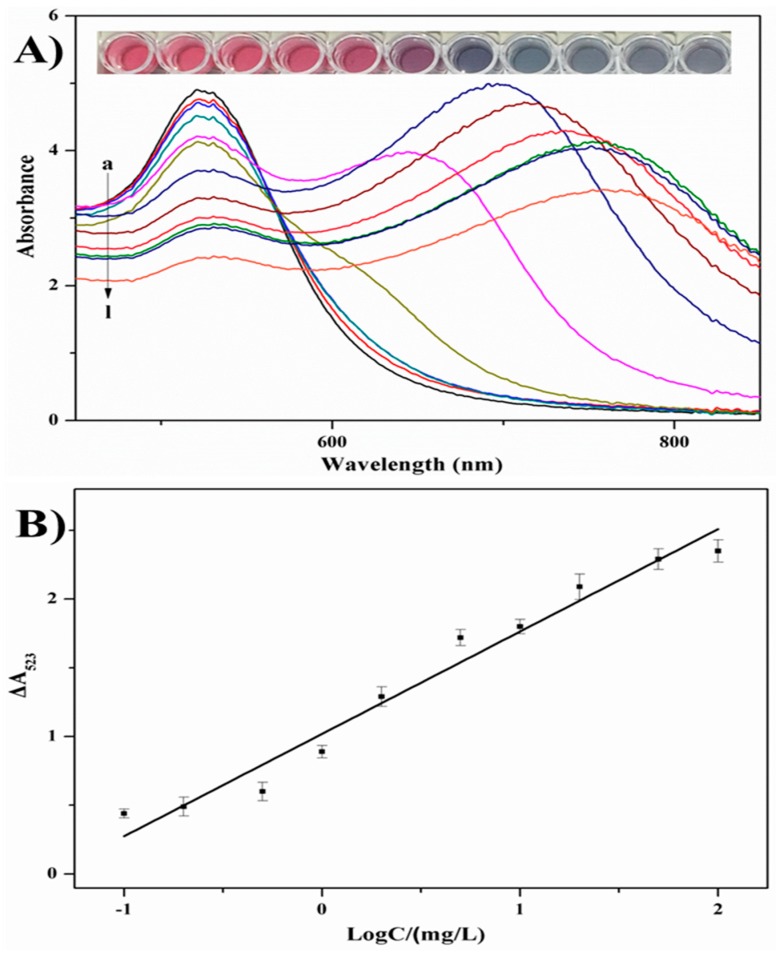
(**A**) UV-Vis spectra and a photograph of (a) AuNP solutions, AuNP solutions with concentrations of chlorsulfuron of (b) 100 mg/L , (c) 50 mg/L, (d) 20 mg/L, (e) 10 mg/L, (f) 5.0 mg/L, (g) 2.0 mg/L, (h) 1.0 mg/L, (i) 0.5 mg/L, (j) 0.2 mg/L, (k) 0.1 mg/L (l) 0 mg/L in the presence of 20 mg/L acetamiprid and (**B**) standard calibration curve of the absorbance change of AuNPs at 523 nm (Δ*A_523_*) against chlorsulfuron concentration.

To determine the sensitivity and linear range of this anti-aggregation sensing strategy, solutions with different chlorsulfuron concentrations (0.1–100 mg/L) were added to the reaction solution under the optimized conditions. The intensity of the absorption peak at 640 nm decreased and the intensity of the surface plasmon resonance absorption of AuNPs at 523 nm increased (Figure 6a). An obvious color change from blue (purple) to Bordeaux red occurred. 

In addition, the linearity was evaluated with different chlorsulfuron concentrations using the equation Δ*A_523_* = *A*_blank_ − *A*_sample_, where *A*_blank_ is the absorbance recorded at 523 nm without any chlorsulfuron, and *A*_sample_ is the absorbance recorded at 523 nm with chlorsulfuron. The analytical signals (Δ*A_523_*) were linearly related to the logarithm of different chlorsulfuron concentrations (0.1–100 mg/L). The calibration curve of Δ*A_523_* versus chlorsulfuron concentration (Figure 6b) gave a linear relationship (*y* = 0.732*x* + 1.03), with a correlation coefficient (*R*^2^) of 0.987. For a signal-to-noise ratio of three, the limit of detection was 0.025 mg/L. The analytical performance of this colorimetric sensing method was compared with other chlorsulfuron detection methods (Table 1). Compared with other methods, the colorimetric assay established in this work is simpler, more rapid, and more economical, with no need for sophisticated instruments or time-consuming treatment procedures.

### 3.5. Selectivity

**Figure 7:**
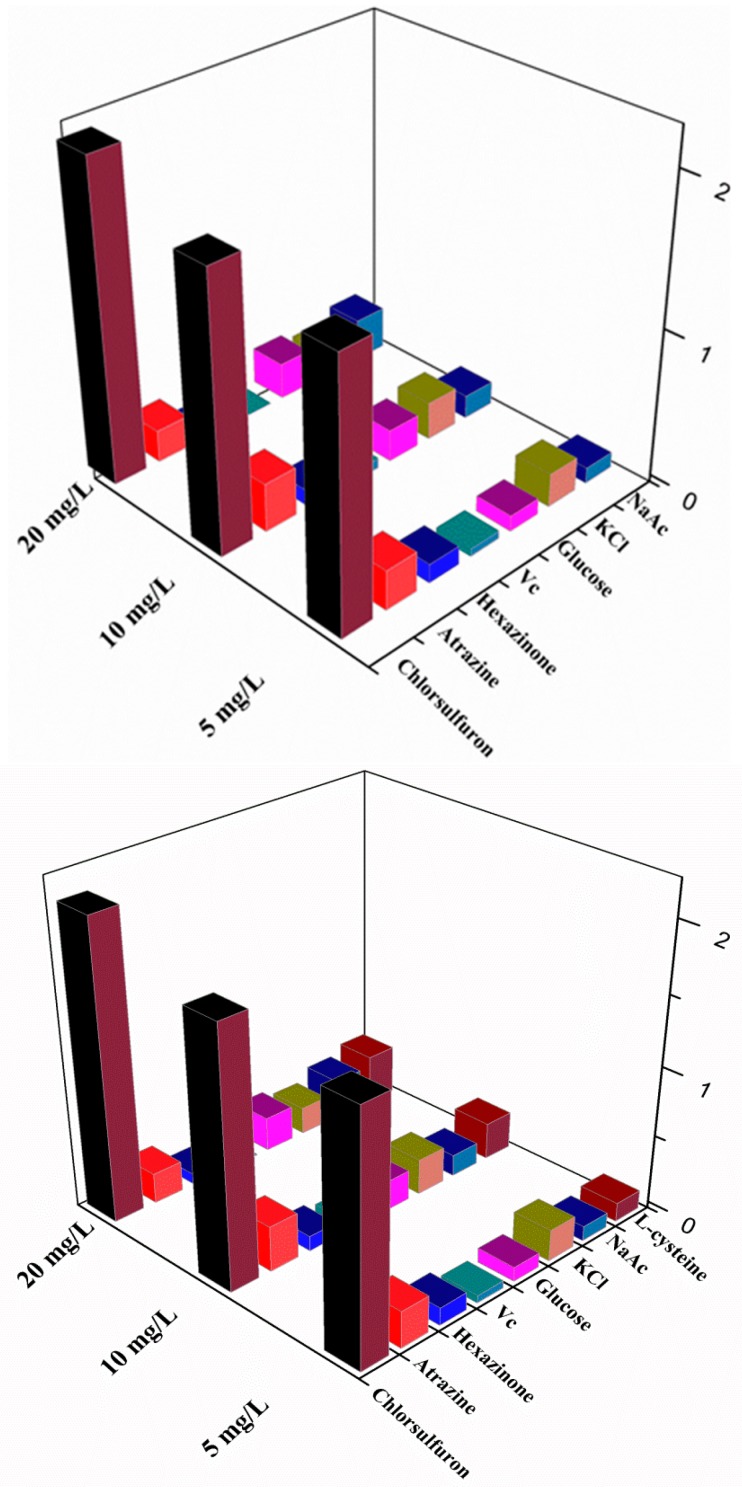
Absorption change Δ*A_523_* of AuNPs in the presence of chlorsulfuron and other interfering substances.

To assess the selectivity of this method for chlorsulfuron, the analytical signals of some common potential interfering substances (atrazine, hexazinone, glucose, l-cysteine, vitamin C, Na^+^, and Mg^2+^) were determined at 5, 10, and 20 mg/L. No obvious signal changes were observed in the presence of atrazine, hexazinone, glucose, l-cysteine, vitamin C, Na^+^, and Mg^2+^ (Figure 7). Only chlorsulfuron showed anti-aggregation and prevented a color change of the AuNPs from Bordeaux red to blue. However, heavy metal ions such as Pb^2+^ and Hg^2+^ could induce aggregation of AuNPs in a similar manner to acetamiprid, which would affect the quantitative analysis of chlorsulfuron. Consequently, it is very important to use EDTA to remove heavy metal ions from agricultural irrigation water samples before analysis using the developed method. 

### 3.6. Application to Agricultural Irrigation Water Samples

The analytical performance of this colorimetric sensing system in a practical application was tested. Tap water, river water, and well water samples were treated with 1 mM EDTA and then spiked with standard solutions. Next, the chlorsulfuron concentrations were determined using the developed method. The recoveries of 0.5, 1.0, and 5.0 mg/L chlorsulfuron from the spiked environmental water samples ranged from 76.3% to 94.2%, and the relative standard deviations ranged from 3.62% to 7.34% (Table 2). These results suggest that the developed colorimetric assay could be used to monitor chlorsulfuron in environmental water samples.

## 4. Conclusions

A simple, rapid, and sensitive colorimetric detection method was developed for chlorsulfuron in agricultural irrigation water based on the anti-aggregation of AuNPs. The aggregation of AuNPs was induced by acetamiprid with an accompanying distinct color change from Bordeaux red to blue. However, strong hydrogen bonding between chlorsulfuron and acetamiprid could inhibit AuNP aggregation. The analytical signals (Δ*A_523_*) were linearly related to the logarithms of the chlorsulfuron concentrations (0.1–100 mg/L). The detection limit of chlorsulfuron was 0.025 mg/L (signal-to-noise ratio of 3). In addition, this colorimetric sensing assay was simple and rapid, without the need for any sophisticated instruments or complex experimental steps. The high sensitivity and good recoveries achieved indicate the colorimetric method may be used to monitor chlorsulfuron residues in environmental water samples.

## Figures and Tables

**Table 1:** Comparison of the linear ranges and detection limits of different methods. LOD: Limit of Detection; HPLC-CAD: Liquid Chromatography-Charged Aerosol Detector; HPLC-UV: Liquid Chromatography-Ultraviolet Detector.

Methods	Linear Range (mg/L)	LOD (mg/L)	R^2^	Matrices	Reference
HPLC-CAD	0.01–1.0	0.01–0.05	0.999	Grain, straw, green plant	[6]
HPLC-UV	0.02–5	0.08	0.999	Soil	[7]
Fluorescence detection	0.01–1.0	0.01	0.999	Environmental water	[9]
Electrochemical Immunoassay	-	0.005	-	Environmental water	[11]
Anti-aggregation-AuNPs based UV–Vis detection	0.1–100	0.05	0.957	Irrigation water	This work

**Table 2:** Recoveries of chlorsulfuron in water samples (*n* = 3). RSD: Relative Standard Deviations.

Samples	Spiked (mg/L)	Found (mg/L)	Recovery (%)	RSD (%)	LOD (mg/L)
Tap water	0	-	-	-	0.125
	0.5	0.421	84.1	4.25	
	1.0	0.817	81.7	3.62	
	5.0	4.52	90.4	6.47	
Well water	0	-	-	-	0.25
	0.5	0.382	76.3	7.34	
	1.0	0.785	78.5	4.19	
	5.0	4.71	94.2	5.62	
